# Evidence-based guideline implementation of quality assurance and quality control procedures in the Saudi National Mental Health Survey

**DOI:** 10.1186/s13033-017-0164-0

**Published:** 2017-09-25

**Authors:** Sanaa Hyder, Lisa Bilal, Luma Akkad, Yu-chieh Lin, AbdulHameed Al-Habeeb, Abdullah Al-Subaie, Mona Shahab, Abdulrahman Binmuammar, Feda Al‐Tuwaijr, Noha Kattan, Yasmin Altwaijri

**Affiliations:** 1 Biostatistics, Epidemiology and Scientific Computing Department, King Faisal Specialist Hospital and Research Centre, MBC 03, PO Box 3354, Riyadh, 11211 Saudi Arabia; 2 King Faisal Foundation, Riyadh, Saudi Arabia; 3 Survey Research Center, Institute for Social Research, University of Michigan, MI Ann Arbor, USA; 4 Mental Health and Social Services, Ministry of Health, Riyadh, Saudi Arabia; 5 Edrak Medical Center, Riyadh, Saudi Arabia; 6 Leiden University Medical Center, Leiden University, Leiden, The Netherlands; 7 Corporate Planning, Saudi Aramco, Dhahran, Saudi Arabia; 8 Vision Realization Office, General Sports Authority, Riyadh, Saudi Arabia

**Keywords:** Quality control, Mental health, Survey, Epidemiology, Survey methodology

## Abstract

**Background:**

The World Mental Health surveys have been known to apply high standards of quality control, but few studies have been published to document this. Furthermore, the effectiveness of quality control has rarely been reported in the Middle East.

**Case presentation:**

The focus of this paper was to highlight the implementation of quality control procedures in the Saudi National Mental Health Survey under the World Mental Health Survey Consortium. The paper summarizes the guidelines implemented for the various phases of survey quality control—the quality assurance procedures, the quality control procedures and the quality control appraisal components—as per previously prescribed recommendations in literature.

**Conclusions:**

Survey quality management is a process and not reducible to a single event. Midstream corrections are warranted by detecting problems and intervening appropriately. The Saudi National Mental Health Survey implemented such procedures through continuous quality improvement.

## Background

Quality assurance refers to the planned procedures and activities an organization uses to ensure that the study meets quality requirements [1, 2]. Planned efforts to monitor, verify and analyze the quality of data as it is being collected are regarded as quality control [1]. Quality assurance anticipates problems as opposed to quality control, which responds to observed problems [3]. Cumulatively, such quality-related procedures enhance the reliability and validity of a survey [4].

The World Mental Health (WMH) Survey Initiative was launched to bridge the gap between the growing mental health global epidemic and the availability of appropriate resources [5]. Saudi Arabia joined in this effort and conducted the Saudi National Mental Health Survey (SNMHS). The SNMHS is a community-based epidemiological study conducted under the patronage of the King Salman Center for Disability Research, Saudi Arabia.

The main objective of the SNMHS is to estimate the psychiatric morbidity in different regions of Kingdom of Saudi Arabia (KSA), and the magnitude of disability caused by it. The project attained a sample of 4000 respondents, males and females between the ages of 15 and 65, who were selected randomly from Saudi households. This sample covered all the different regions in the Kingdom. Face-to-face computer-administered interviews were conducted in the homes of the participants.

All WMH surveys apply high standards of quality control, but few studies have been published to document this [6–9]. Furthermore, the effectiveness of quality control has rarely been reported in the Middle East. This may possibly be because the demand for substantive publications forces periodicals to adopt restrictive policies pertaining to article length, which overrules the inclusion of data about the “backstage” of health surveys [8]. It is crucial to give attention to quality now more than ever because of the increasing demand to collect more quality data at a lower cost and its implications for health policies [10]. The focus of the present paper is to highlight the implementation of QC procedures in a large household survey like the SNMHS and underline its substantial and effective role in conducting survey research in KSA. The sections below summarize the various phases of quality control—the quality assurance procedures, the quality control procedures and the quality control appraisal components—in the Saudi National Mental Health Survey as per previously prescribed recommendations in literature. The quality phases below were outlined based on the elements of survey process quality management, which allows users to assess the quality of processes throughout the survey lifecycle [1, 8, 11–13].

## Case presentation

### Quality assurance

Literature suggests setting up a comprehensive system of quality assurance at the start of fieldwork, including several simultaneous mechanisms [10, 14]. According to quality standards and assurance procedures in literature [1, 10], the following criteria were established to minimize survey-related errors. These criteria are ideally established during the planning phase of a survey [3].

### Operational team

The wide geographic dispersion of the sample in the SNMHS, the long duration of the survey, and the large sample size mandated several supervisory levels. The SNMHS central team comprised of various specialists including the principal investigators, project manager, project coordinator, research assistants, the data manager, the QC staff, the team of verifiers (part of QC), and the IT (Information Technology) helpdesk staff. Additionally, the field team comprised of interviewers, supervisors, field managers and field coordinators, who were trained and WMH certified to conduct household interviews with the selected respondents, using laptops and specialized software to reduce the data entry error rate and improve accuracy. Finally, a team of specialists at the University of Michigan Ann Arbor and Harvard University formed a crucial part of the SNMHS; these specialists worked as partners in the project, who provided long distance support and consulted regularly throughout the project, as the leaders in this subject and having experience in conducting WMH surveys in over 30 countries around the world.

**Fig. 1:**
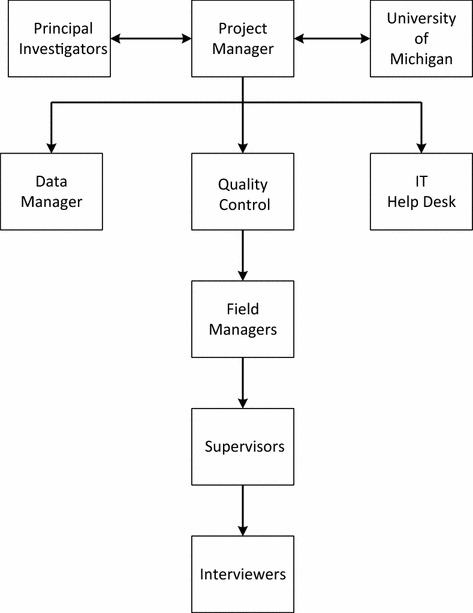
Coordination between the SNMHS team (goes here)

In terms of responsibilities, the central team maintained regular contact with the survey field team to ensure the protocols were being followed. The program manager and project coordinators supervised the progress of fieldwork on a daily basis. The QC staff monitored the progress of the survey activities in real-time on a daily basis using sophisticated software tailor-made for the project. A team of verifiers called respondents over the phone and checked for any interview falsification (for a detailed overview, see *‘Verification’* under *Quality Monitoring and Control).* The IT helpdesk team, available at all times, addressed various issues (technical, study protocol-related or personal) faced by the interviewers in the field. With regard to the field team, the field managers headed a team of field supervisors that oversaw the interviewers’ work at the local level. A field coordinator was on call during data collection. Finally, the University of Michigan Ann Arbor staff provided overall support to the project, including: developing training sessions for interviewers, programming the questionnaire into a computer format, and managing the QC programs. As for the Harvard University staff, they provided support for data management, diagnostic algorithms, and data analysis. See Fig. 1 for an illustration of the coordination between SNMHS team.

Overall, the SNMHS quality protocols adhered to the recommendations in literature, as the project embedded diverse and talented staff members, during the early stages of the project, who were trained to handle quality concerns, thereby ensuring the survey was reliably implemented [3, 10]. The formation and functions of the SNMHS staff were also established so as to ensure that they pay careful attention to the quality of survey implementation and monitor it in real-time, ensuring problems were addressed while the survey was in progress.

### Sampling

The SNMHS used a stratified multistage probability proportionate to size sample design based upon the 2010 estimated population by the General Authority for Statistics, Saudi Arabia [15]. These sampling methods were consistent with previous WMH survey studies, which used multistage sample designs [6, 8]. The SNMHS relied on its field team to update any inconsistent information on the sampling frame from the 2010 census. Field staff was trained on how to identify a household in a manner that is consistent with the sampling frame construction. A household referred to an estate unit containing one or more rooms that was originally intended for domestic use or to be occupied by a single family; more than one family could reside in the household. Different types of households included villas, apartments, and tents [15]. Moreover, to aid in the process of locating households, all field staff were equipped with global positioning system (GPS) devices.

### Translation

The SNMHS used the WHO Composite International Diagnostic Interview (CIDI) Version 3.0. The CIDI 3.0 is a structured interview that generates diagnoses for a wide range of mental-health disorders using the diagnostic statistical manual 4 (DSM-IV) and the International Classification of Diseases (ICD-10) criteria [9, 16]. CIDI 3.0 was translated and adapted by a team of Saudi physicians and translators, and revised by an expert panel following the TRADP (translation, review, adjudication, pretesting, and documentation) approach [17]. Details of the Saudi adaptation of CIDI 3.0 will be published in the future.

The 500-page paper and pencil administered questionnaire was fully programmed into a computerized version (CAPI: Computer Assisted Personal Interview) with an audio-assisted component (ACASI: Audio Computer Assisted Self Interview) for potentially sensitive questions. This allowed the interviewers to conduct the interview in the households using laptops provided by the project. Using the computerized version of the interview reduced errors, eliminated the data entry step, and allowed for close monitoring and quality control of the fieldwork [13].

Following translation of the CIDI 3.0, cognitive interviews were conducted to identify any problematic questions in relation to the different response process stages (e.g. comprehension, recall, and judgment). Findings from this pretest suggested that different types of cognitive probes elicited different types of sensitive feedback. Additionally, the pilot study revealed that the original questionnaire was long. Thus, many noncore questions were eliminated to shorten the interview length. The US and KSA team spent an entire year working on revamping the questionnaire, testing and re-testing, and administering the CIDI on a clinical and non-clinical sample.

### Training

Training of a survey team is key to quality and should be provided at all levels i.e. the interviewers, supervisors, trainers, as well as the central team overseeing the process nationally [10, 18]. In the context of the SNMHS, the principal investigators and key members attended 2 month long training sessions in the Institute of Survey Research at the University of Michigan Ann Arbor, USA. A train-the-trainer session was also conducted in Jeddah, Saudi Arabia, in collaboration with visiting experts from the World Mental Health Data Collection Coordination Center at the University of Michigan, Ann Arbor. These training sessions lasted 6 days, and were designed to prepare the interviewer supervisors to train and monitor interviewer performance, as well as to manage data collection and data processing in Saudi Arabia. Through these sessions, the research teams obtained information and materials necessary to train their own interviewing staff using consistent standardized procedures.

Although the SNMHS relied on a local research company in the initial phase of the study, the principle investigators later decided to take control over the fieldwork operations and proceeded to independently recruit the field teams, comprising of interviewers, supervisors and field managers. Field members were recruited if they met the requirement of minimal education level (high school graduates), good communication skills, passing certification before commencing fieldwork, and availability on weekdays, weekends and national holidays.

The SNMHS central team trainers, conducted a total of 7 supervisor and interviewer training courses in different regions of Saudi Arabia. In order to obtain high-quality data collection procedures, using standardized protocols, all trained supervisors and interviewers went through a 2-week certification course for the CIDI 3.0 instrument prior to beginning fieldwork [1]. Similar training sessions and certification tests were implemented by previous studies that undertook the WMH survey initiative [6, 7].

The interviewer training covered various topics such as general interviewing techniques, CIDI-specific training, sample management system training, and fieldwork training, which included a presentation on how to find and approach sample houses. All sessions were conducted in person, and all interviewers were provided with a comprehensive study manual translated in Arabic. In addition, the interviewers were tested on computer hardware and software use, including the use of CAPI questionnaire administration. Overall, the content of the training sessions was in sync with previous studies [8].

The survey had a full time IT helpdesk staff, which was on-call throughout the data collection phase to provide live technical support to the interviewers. The helpdesk staff received web-based lectures by the University of Michigan Ann Arbor, and some members visited Ann Arbor for additional training. Helpdesk members also trained with the KSA team learning the study protocol, and the sample management system, SurveyTrak.^1^

The SNMHS also conducted two booster sessions for the field team, after specified interims in the fieldwork phase, where they reviewed various aspects of data collection and focused on issues that were found to be complex or difficult, and were reminded of guidelines that were not being adhered to sufficiently by interviewers [10].

### Pretesting

#### Pilot study

Conducting a pilot survey which tests the entire survey protocol from start to finish on a small scale is a significant element of a quality assurance strategy [3]. The procedures, instruments, administrative and logical aspects of the planned larger SNMHS were pretested to maximize survey data quality. The SNMHS pilot study was conducted in 2011 by 19 physicians from the MOH, who underwent a 6-day intensive course in Riyadh, Saudi Arabia, which was conducted by experts from the University of Michigan Ann Arbor, to become CIDI certified interviewers. A list of households from the neighborhoods in Riyadh was divided among the interviewers. The interviewers visited the households where they conducted the CIDI interview after randomly selecting 1 male and 1 female respondent from each household. A total of 74 interviews were completed successfully (response rate 81.6%). The length of the instrument was found to be longer than expected (3.5 h on average) and suggested the need to reduce it. Some questions needed rewording and more clarity. Largely, the success of the SNMHS pilot test indicated its readiness to be implemented on a national scale. Similar methodologies for finalizing an instrument for a survey have been employed by previous studies [8]. Further details of the pilot study can be found elsewhere [19].

#### Cognitive interviews

As mentioned earlier, cognitive interviews were designed to pretest the adapted Saudi version of the CIDI questionnaire. The cognitive interviews aimed to investigate feedback differences in cognitive probes designed to pretest for question sensitivity. The study included Saudi males and females of different age groups and educational backgrounds. The cognitive interviews were conducted at the clinic where the patients (with a history of mental disorder) were recruited from, at the patients’ houses, as well as at KFSH&RC, Riyadh. While the interviewer conducted the interview and took some notes, an observer made additional notes. The application of cognitive interviews in such situations was essential, since a well-designed question in the original language and intended culture could be inappropriate in the target culture. Results of this pretest will be published in the future.

### Data capture

In computer-assisted personal interviews, the data is entered as the interview is in progress with checks built into ensure correct application of the interview with all skip and branching rules to reduce data entry error and ensure data consistency [10, 13]. The SNMHS used CAPI to administer the CIDI instrument and interviewers were instructed to send the data daily to a central server using the sample management system.

Using such computerized data collection methods has several advantages such as: reducing item missing data, getting timely data, and collecting process data or paradata [20]. Broadly, paradata referred to process data such as call records, interviewer observations, time stamps and other useful data [13, 21].

However, some of the disadvantages include laptop or software error while the interview is being administered, data not stored properly, and laptops being stolen. While these situations occurred, they were infrequent and the SNMHS helpdesk team was able to remedy them promptly, consistent with how previous studies managed these type of issues [13].

### Quality audits

Quality Audit is defined as the systematic examination of the quality system of a survey by an internal or external quality auditor or team [1]. In the context of SNMHS, a team at the University of Michigan Ann Arbor supervised all the QC-related activities of the survey and externally audited them. Within the SNMHS central team, a team of internal auditors (known as verifiers) verified a random subsample of the completed interviews by re-contacting specific respondents and re-administering a small subset of questions. Moreover, in the field, a team of supervisors observed the performance of their assigned interviewers to ensure they were following protocol and meeting certain benchmarks. Another kind of audit was conducted annually by the Office of Research Affairs at KFSH&RC, where all the consent forms collected from the respondents were scrutinized and reviewed to ensure that the study was following ethical standards.

## Quality monitoring and control

The following list of tools was used by the QC staff to monitor the quality of the fieldwork.

### Quality control cube

Previous literature has exhaustively praised tools that allow rapid analysis of CAPI interviews and detect signs of low interview quality [6, 13, 18, 21–23]. Consistent with this recommendation, the University of Michigan Ann Arbor survey team designed an on-line analytical processing (OLAP) cube to be used by the local QC team in KSA that pulls keystroke data, questionnaire data and sample management data (SurveyTrak), and processes them into quality indicators that are displayed in Excel with Excel pivot functionality.

The cube grouped the indicators into three levels of key performance depending on need for intervention. Indicators of quality were used to evaluate contextual factors that affected the quality of a survey [1, 10, 13]. Level 1 indicators flagged any interview with possible deviation in survey protocol which required immediate investigation and were reviewed daily by the QC staff. An example of this type of indicator was when an interview was flagged for having one or more pauses which were over the cut-off limit of 10 min. A member of the QC staff then traced the keystroke file for the flagged interview to look for a justification for the long pause. In the context of the SNMHS, the interviewer usually left a note saying they took a short break to pray (as Muslims pray five times each day). Given the note explained the pause, the issue was marked resolved. However, when an explanation was not found, the QC member contacted the fieldwork supervisor and requested appropriate action to be taken. Similar investigation procedures have been recorded in literature [14, 18, 24–28].

Level 2 and Level 3 indicators showed percentages or averages which were used to rank interviewers based on priority for investigation. Over time, these type of indicators depicted erroneous patterns that allowed the QC staff to detect deviations in an interviewer’s performance. These indicators were reviewed weekly as the rates needed to be accumulated before indicating outliers. Regardless of the indicator level, all flagged cases were investigated, justified and documented. Table 1 gives some examples of indicators compiled by the cube. Additional details about the cube can be found elsewhere [23].

**Table 1:** SNMHS Quality Indicators

Cube	Indicators
Level 1	An interview with question read very quickly or skipped (under 1 s)
	Long pause (> 10 min)
	Short interview (< 30 min)
	Failed verification
Level 2	Rate of unable to verify cases
	Rate of interviews detected with mental health disorders (Prevalence Rate)
	Average interview length
	Rate of saliva refusal
Level 3	Rate of household with no eligible male
	Rate of household with no eligible female

### Webtrak

It is important to capture data accurately and in a timely manner as this ensures immediate local data entry, quality check and central coordination [10, 13, 20]. For this purpose, WebTrak,^2^ a web searchable tool that pulls data from the sample management system, verification and evaluation forms was used extensively by the SNMHS, especially by the project manager, QC staff, field staff and the IT helpdesk. WebTrak helped the survey team monitor samples, and access specific details by using its varied search function. The QC staff specifically used it to investigate flagged and problematic interviews/interviewers. This type of inspecting included checking ‘call notes’ left by the interviewer or determining details related to specific sections of the interview (e.g., ACASI), to justify any inconsistencies in a specific interview. The program also displays reports, which were generated and updated on a daily basis to monitor response rates, cooperation rates, refusal rates, interview length, etc. At large, WebTrak gave a holistic account of the fieldwork, especially in terms of paradata. Overall, programs like SurveyTrak and WebTrak prevented many types of interviewer-related fraud (e.g. interviewers making up/faking interviews). As noted in previous studies, when specialized softwares were not employed, interviewer-related fraud resulted in discarding a number of interviews, in turn hindering the fieldwork procedures [7, 8].

### Verification

Verifications are mainly conducted to detect interview falsifications [1, 20, 24]. The SNMHS verification team carried out phone verifications as well as face-to-face verifications [8, 18]. Face-to-face verifications were conducted when there was no available contact number or there was no contact with the household (either by call-attempts or because its eligibility was unknown). Verifications were promptly conducted after an interview was finalized. A finalized interview was an interview that received a final code to indicate its status, and was flagged for verification by the related softwares.

Verifiers used a script when calling households, which identified the verifier and explained why they were calling. All verifications were usually completed within 2 weeks of an interview being flagged and the responses were recorded on WebTrak. If an interview was coded “Unable to Verify” the verifier manually flagged a different completed interview by the same interviewer for verification.

The QC team manually flagged additional lines for verification in several instances, such as when: (i) the interview length was less than 35 min (average interview length in the SNMHS was 129.42 min) (ii) three or more interviews were conducted by an interviewer in 1 day (iv) an interviewer had high rates of interviews where respondents refused to give saliva (the SNMHS collected saliva samples to determine genetic psychiatric morbidity) (v) an interviewer had low eligibility rates for male or female respondents, or (vi) any other reason, as seen required [20]. A verifier explained the details of a failed verification using the “additional comments” section in the verification form on WebTrak. For failed verifications, the QC staff immediately took the appropriate action to intervene in the interviewer’s performance. Moreover, the QC manager supervised all verifications, reviewed WebTrak verification reports, discussed cases with the verifiers and investigated specific interviews by contacting the field staff.

### Evaluation

Interviewers need to be evaluated to determine if they are employing their interviewing skills effectively and if they require any support [10, 18]. For this reason, the fieldwork supervisors accompanied all interviewers into the field and filled out evaluation forms. This feedback was recorded on WebTrak, which was accessed by the QC team as well as specialists at the University of Michigan, Ann Arbor [20].

These evaluations allowed the supervisors to track the interviewers’ progress, compare their performance with that of their peers, identify where they were making errors and advise them accordingly; this in turn helped improve interviewers’ ability, morale and productivity. Such a practice wherein the supervisors evaluate the interviewers’ performance, was consistent with previous studies [6, 7, 13, 18, 24, 28, 29]. These evaluations were conducted on a periodic basis and focused on conformity with the interviewing conventions and guidelines, and identification of interviewer-questionnaire interface problems. In case an interviewer did not ‘pass’ their evaluation, they were retrained for the components in which they made errors. They were then accompanied again by their supervisor for the following interview(s) until the supervisor determined they had passed the evaluation.

### Interventions

Literature emphasizes that recommended corrective and preventive actions are important components of survey quality management [1]. Interventions were requested when a problematic pattern was observed across multiple indicators or a change in a specific indicator was observed over time [20, 30]. In the context of SNMHS, these patterns were often linked to primary sampling unit assignments (each administrative area of KSA was divided into several primary sampling units), which varied as the fieldwork team traveled between urban and rural areas for data collection. In these situations, the QC staff had to consider cultural differences within the country before deciding to intervene in an interviewer’s performance.

#### Corrective actions

Based on feedback, some recommended corrective actions for the interviewers’ performance included extended field evaluation, retraining, suspension and verification [20]. (i) Extended evaluation was prescribed for a particular interviewer based on his/her percentages or occurrences of flagged indicators, as derived from the QC cube. Extended evaluation required comparing a particular interviewer’s past versus ongoing performance for the problem indicator, i.e. when he/she conducted interviews in the absence of their supervisor versus interviews conducted in the presence of their supervisor. Extended evaluation was carried out when there was a problematic pattern observed for a combination of indicators. (ii) Retraining involved holding repeat training sessions for the interviewer in question, for specific problem indicators (e.g. percent of ACASI switching to CAPI, and percent of saliva not given) or general interviewing techniques (probing non-response). (iii) Suspension indicated suspending the interviewer for a specific duration, in which the survey team came to a decision regarding the interviewer’s performance. (iv) Verification involved verifying all the interviews conducted by the interviewer in question so as to rule out interviewer cheating and protocol violation.

#### Preventive actions

Some recommended preventive actions for the interviewers’ performance included the supervisor keeping an eye on a particular interviewer’s performance for anomalies, as well as reminding the interviewers to follow the necessary fieldwork protocols using a handbook developed by the QC staff.

### Updates to quality plan

Literature enlists evaluation of the quality management plan as a crucial step in quality monitoring and control, indicating that it is important for a survey team to regularly reconvene to discuss possible updates to their QC plan [1, 13]. In the context of SNMHS, the QC staff held regular meetings to discuss their quality management strategy. These meetings involved deliberating over the effectiveness of the QC softwares, division of duties between the members, and offering recommendations/suggestions that could improve the existing strategy.

## Quality control appraisal

Chiefly in the context of this paper, QC appraisal referred to developing a ‘quality profile’. A quality profile synthesizes information from other sources, documenting survey methodology used throughout the survey, lessons learned and recommendations for improvement, in turn providing users all the information needed to assess data quality [1]. The different sources that contributed to the quality profile of the SNMHS included:

### Documentation of process protocols

Documenting survey implementation methods in a systematic manner in the form of qualitative reports as well as quantitative indicators (e.g. response rates) gives users critical information about the quality of a survey [3, 10, 13]. For this reason, the QC staff developed standard operating procedures (SOPs) for all tasks carried out within the survey including data management, fieldwork protocols, quality control as well as IT procedures. They prepared specialized manuals for verification, helpdesk support, fieldwork and quality control protocols. These manuals elaborated on the procedures and enlisted the different steps needed to carry out related investigations. The QC staff also developed a handbook for interviewers, which elaborated on interviewing protocols.

Another documentation technique employed by the QC staff was the usage of Google spreadsheets. These spreadsheets varied in their function. For instance, the QC members used regional spreadsheets for all the interviewers in a particular region, to record their flagged cases, their justifications, and whether or not the flagged cases were resolved. Similarly, verifiers recorded the outcome of their verification lines on a separate Google spreadsheet. The SNMHS team in collaboration with the University of Michigan Ann Arbor also shared a spreadsheet, which recorded the various interventions prescribed for the interviewers in the field.

It was also useful to produce periodic reports on the results of quality control procedures and the performance of individual operators, as this helped to identify mechanisms that needed improvement [3]. The SNMHS prepared reports based on the feedback it received from some of the respondents about their interviewer’s performance. This report helped to identify what the project was doing right and what needed to be improved in the future within the SNMHS. This report was consistent with previous studies, wherein they emphasized documentation of modifications to study protocol [1]. The SNMHS also prepared annual and bi-annual reports for its funding agencies/sponsors. These reports included the survey’s achievements in a specific duration, challenges faced in that duration, modes of actions for the next phase, the research outputs and an inventory of the budget expended.

Literature also emphasizes using a protocol for data coding and data entry staff, and related outcomes [1]. In this regard, the SNMHS central team employed a QC documentation technique known as the weekly QC checks report [25]. This report was prepared using weekly-updated information generated from a customized database on Microsoft Access 2010. This information highlighted indicators that might concern the QC staff and thus require further investigation. For instance, ‘inconsistent gender’ indicated that the gender of a particular interviewer did not match the gender of the respondent who completed/partially completed a main interview; this was considered an anomaly as the interviewing method was gender-specific. Another instance was ‘interview length’ where the query showed the shortest and longest screeners/main interviews done, which were not necessarily problematic, unless for example, screeners were completed in less 2 min or in more than 30 min; these cases then required investigation. Thus, these reports served as additional measures, ensuring QC was well maintained. The SNMHS received funds from various sponsors to conduct the study; thus, the overall budget of the project and its expenditure were documented and submitted for auditing. As per previously prescribed guidelines, the SNMHS also intends to prepare local and international reports, and executive summaries for policy makers, the public, researchers and other scientific users, in the future [4].

### Feedback

Feedback is an integral part of a formal QC program. It can be verbal or written, and be in the form of reports, tables or graphs of the results and assessments compiled during the inspection process; these were relayed to various levels of staff associated with the survey operation so as to improve quality [3]. An example of this type of feedback as mentioned before, was the feedback obtained from respondents about their interviewer’s performance.

Literature also encourages user feedback [1, 13]. Within the SNMHS, weekly update meetings as well as one-on-one meetings were held between all the members of the SNMHS central team and the project coordinator/manager, to take their feedback into consideration regarding general problems they faced or comments they would like to share about the project’s progress/quality. The project manager also received meeting min from the field manager and weekly reports from the supervisors about fieldwork issues. This exercise was consistent with literature [6, 25]. Furthermore, the field staff comprising of supervisors, field managers and interviewers provided feedback for the services provided by IT helpdesk staff. The SNMHS’ KSA base (i.e. the Biostatistics, Epidemiology, and Scientific Computing Department at KFSH&RC, Riyadh) as well as the organization higher authorities, which house the SNMHS project, also provided feedback to the project coordinator/manager and principal investigators about the project’s progress. Nationally, the sponsors of the SNMHS are expected to provide their feedback about the project’s impact in the future. On an international level, the University of Michigan Ann Arbor gave constant feedback to the SNMHS central team in Riyadh regarding the project’s developments. The many ways in which the SNMHS received feedback ensured that the project constantly updated and improved its quality-related mechanisms. Feedback also helped to maintain strong levels of cohesion and motivation within the project teams [6].

## Limitations and lessons learned

Lessons learned should be documented as they prove useful not only for the study’s coordinating center and national survey studies, but also for researchers and organizations interested in conducting similar studies, especially in Saudi Arabia and in the neighboring countries [1]. As the SNMHS was the first national mental health household survey in KSA, it experienced several challenges. Firstly, the study noted that the quality of the fieldwork team was a recurring obstacle where, it was difficult for the study to obtain a good number of qualified interviewers, supervisors and field managers, especially with experience in the research field. It was difficult to retain skilled interviewers after fieldwork or find interviewers who were able to work full-time; this prevented the survey fieldwork from becoming more efficient and progressing quickly. In some fieldwork phases, some interviewers disrupted the efficiency of quality management by not adhering to professional etiquette of communicating with related authorities (supervisor/field manager/project manager). Secondly, cultural traditions stalled fieldwork in varied ways. The concept of health community projects is not familiar to people in Saudi Arabia; some respondents were wary of the letters provided by the interviewers from the Ministry of Health, Ministry of Interior, and the local police. Some sample household areas were difficult to reach and not safe for female interviewers to be moving about alone. This was problematic as it is not common in Saudi Arabia to see females walking alone in residential streets. Thirdly, a small number of respondents provided information (e.g. presence of a third person) during the interview that was difficult to assess during verifications. Future studies should consider recording (audio/video) their survey interviews, as this would aid quality control activities. However based on these challenges, the booster training sessions and the prospective trainings for field staff comprised of improved and updated content, which trained the field staff to better handle the challenges and be more adept. The course content and material of the training sessions were constantly updated by the project manager to address the challenges that emerged throughout the course of fieldwork.

Prospective studies should consider using computers to conduct all aspects of their survey, and especially think about employing computer-assisted instruments (e.g. CAPI), as their success has been recorded in literature [6, 8]. In line with literature, they should also document various aspects of a survey as documentation of methodologies, feedback and progress is important for the purpose of review, assessment and follow-up [1, 3]. Finally, developing a strong QC team, alongside adopting and employing well-established and efficient QC tools to manage quality procedures should be a high priority.

## Conclusion

The SNMHS is the first community—wide survey of psychiatric morbidity that was conducted in a nationally representative sample of the Saudi population. The assessment of quality procedures on an ongoing basis during the course of the survey was essential, where midstream corrections were warranted by detecting concerns and intervening appropriately [10–12]. The SNMHS implemented such procedures through continuous quality improvement, thereby instilling confidence in the data it yielded. This paper is consistent with literature which argues that quality management is a process and not reducible to a single event [10, 13]. The quality procedures implemented by the SNMHS are altogether one of its greatest achievements. The SNMHS hopes that its findings will better serve the country’s mental health needs and guide health policy-makers to implement preventative measures and provide appropriate care to the public.

## Abbreviations

WMH: world mental health
SNMHS: Saudi national mental health survey
MOH: ministry of health, Saudi Arabia
KFSH&RC: king faisal specialist hospital and research centre—Riyadh
WHO: world health organization
KSA: kingdom of Saudi Arabia
QC: quality control
IT: information technology
GPS: global positioning system
CIDI: composite international diagnostic interview
DSM-IV: diagnostic statistical manual 4
ICD: international classification of diseases
TRADP: translation, review, adjudication, pretesting, and documentation
CAPI: computer assisted personal interview
ACASI: audio computer assisted self interview
OLAP: on-line analytical processing
SOP: standard operating procedures

## Footnotes

1 SurveyTrak is an in-house proprietary sample management system designed by the Survey Research Operations, Survey Research Center, University of Michigan, Ann Arbor.

2 WebTrak is an in-house proprietary reporting tool designed by the Survey Research Operations, Survey Research Center, University of Michigan, Ann Arbor.
